# Effect of Increasing the Methionine Level and Reducing the Threonine Level in the Diet of Fast-Growing Rabbits

**DOI:** 10.3390/ani13091471

**Published:** 2023-04-26

**Authors:** Pablo Jesús Marín-García, Luís Ródenas, Eugenio Martínez-Paredes, Vicente Javier Moya, María Cambra-López, Enrique Blas, Juan José Pascual

**Affiliations:** 1 Department of Animal Production and Health, Veterinary Public Health and Food Science and Technology (PASAPTA), Facultad de Veterinaria, Universidad Cardenal Herrera-CEU, CEU Universities, 46113 Valencia, Spain; 2 Institute for Animal Science and Technology, Universitat Politècnica de València, Camino de Vera s/n, 46022 Valencia, Spain

**Keywords:** rabbit, growth rate, protein, amino acids, retention, digestibility

## Abstract

**Simple Summary:**

The use of diets with optimal levels of amino acids in animal production will cause the animal’s productive traits to be better, to have less contamination, etc. This work compares two diets: Diet MMM, with an amino acid level according to the current recommendations (M = medium levels of lysine, methionine, and threonine, respectively), and Diet MHL, with higher levels of lysine (H), medium levels of methionine, and lower levels of threonine, which improved protein utilization in a previous experiment. This work analyzes the effects of this novel combination on performance, digestibility, and retention. Finally, it is concluded that this new combination, which reduced the excretion of urea, improves the growth rate, the feed conversion ratio, and the retention of the main nutrients in fast-growing rabbits.

**Abstract:**

The main aim of this work was to evaluate a novel combination of the first limiting amino acids (lysine, methionine, and threonine) in fast-growing rabbits (combination MHL, shown to minimise levels of plasmatic urea nitrogen in previous research, medium level of lysine, high level of methionine, and low level of threonine) and compare it with current recommendations (combination MMM, medium level of all amino acids). A total of 165 weaned rabbits (28 d old) from a paternal line selected for growth rate were used in the growth trial. The effect of the diet on apparent fecal digestibility, as well as the apparent and true ileal digestibility, were studied. Nutrient retention was also determined. Although no differences in digestibility between diets were observed, animals fed with Diet MHL improved the global average daily gain (+2.3 g/d; *p* = 0.0482) and feed conversion ratio (−0.10; *p* = 0.0229). Animals fed with Diet MMM reduced the protein-to-energy ratio retained (*p* = 0.0086). In conclusion, Diet MHL promoted an improvement in growth traits in a paternal line. Consequently, we propose its levels of 6.4, 5.4, and 5.0 g/kg DM of true ileal digestible lysine, methionine, and threonine, respectively.

## 1. Introduction

In growing rabbits, a selection for feed efficiency has been carried out indirectly in paternal lines by selecting an average daily gain (ADG), as there is a genetic negative correlation between these two traits [1,2]. Considering the expected genetic progress in these paternal lines (0.45 g/d per generation and two generations per year; [3]), over the last 20 years, the ADG and requirements of these rabbits may have increased by 40%, and there are currently animals with high ADG. However, since the onset of epizootic illness enteropathy in rabbits, the dietary protein content in commercial diets has been reduced [4]. There is a problem that this work wants to address; the use of the current diets in growing rabbits from paternal lines could be affecting the expression of their genetic potential and, therefore, the correct elaboration of the genetic rankings.

Recently, Marín-García et al. (2020a) [5] observed that, although rabbits with ADG above 50 g/d improved their feed conversion ratio (FCR) compared to animals showing lower ADG, they had lower protein and higher energy retention. Carabaño et al. (2009) [6] reported that dietary protein levels around 140 g/kg did not impair growth performance of growing rabbits with ADG up to 55 g per day, if digestible protein (DP):digestible energy (DE) (DP:DE) is circa. 9.5 and the amino acid supply is correct. However, the higher ADG of the current fast-growing animals and their lower protein retention might suggest the existence of some limiting amino acid when such diets are used in animals with high growth rates. Subsequently, Marín-García et al. (2020b) [7] observed that the plasma urea nitrogen (PUN) level, which corresponds to the amount of nitrogen in the form of urea circulating in the bloodstream, could be an adequate indicator to detect amino acid imbalances in growing rabbit diets. The use of PUN as a bioindicator of amino acid imbalances was consequently validated.

Lysine, sulphur amino acids (methionine and cysteine), and threonine have been considered the first three limiting amino acids in rabbits, and their requirements for growing rabbits are considered well-established [8]. For this reason, using the PUN technique, Marín-García et al. (2020c) [9] tested three dietary levels [M, medium according to current recommendations [8]; H, high, +15% of these recommendations; and L, low, –15%)] of these three limiting amino acids (lysine, methionine, and threonine) in a 3 × 3 × 3 factorial design. Their results established an amino acid combination that minimised the PUN levels in growing rabbits at 48 d of age (MHL for the lysine, methionine, and threonine, respectively) with respect to the current recommendations (MMM).

On the other hand, feed formulation programmes based on the amount of ileal digestible amino acids provide more precise requirements compared to those based on total dietary amino acids. Therefore, it would be advisable to have the ileal digestible amino acids content of the main raw materials, as well as specific animal-related ileal digestible amino acid requirements. In rabbits, information on the nutritional value of raw materials at that level is available [10,11], but there is little information on ileal digestible amino acid requirements [6], especially compared with other monogastric species. Dietary amino acid requirements were initially proposed by Lebas (1989) [12] and recently updated by de Blas and González-Mateos (2010) [8]. Nevertheless, current amino acid recommendations are mainly expressed as total or apparent fecal digestible contents. An apparent ileal digestibility can better describe the amount of nitrogen and amino acids that are absorbed in the small intestine and are available for animals, but they are underestimated because part of the nitrogen and amino acids in ileal digesta do not come from the diet. This part corresponds to the endogenous losses, and their correction results in their true ileal digestibility value. Providing information on the nutritional requirements of animals in terms of true ileal digestibility is a necessary step towards precision nutrition. Furthermore, rabbits have certain peculiarities related to their feeding behaviour [13], such as the caecotrophy contribution [14] or the existence of a great caecum [15]. This fact hinders the proper evaluation of the protein requirements of growing rabbits.

The aim of this work was to evaluate a novel combination of the first limiting amino acids (lysine, methionine, and threonine) in fast-growing rabbits (combination MHL) shown to minimise levels of PUN by Marín-García et al. (2020c) [9] and compare it with current recommendations (combination MMM). To this end, a growth performance trial was conducted to compare both amino acid combinations (MMM vs. MHL) in growing rabbit diets. A nutrient digestibility and retention assay was also carried out. Evaluating both diets at a true ileal level will contribute to increasing the knowledge of protein requirements in growing rabbits.

## 2. Materials and Methods

The experimental procedure was approved by the Animal Welfare Ethics Committee of the Universitat Politècnica de València and carried out following the recommendations of the European Group on Rabbit Nutrition [16] and Spanish Royal Decree 53/2013 on the protection of animals used for scientific purposes.

### 2.1. Animals

One hundred and sixty-five (28-day-old) rabbits from the R line were used. Line R was obtained after two generations of randomly mating from a pool of animals of three commercial paternal lines [17] and then selected by ADG in the growing period during 38 generations. This paternal line is characterised by a high growth rate during the growing period and was developed at the Institute of Animal Science and Technology of the Universitat Politècnica de València.

### 2.2. Experimental Diets

Table A1 (Appendix A) shows the ingredients and chemical composition of the experimental diets used in this work. The basal mixture was formulated according to the current recommendations [8] except for the three most limiting amino acids (lysine, methionine acids, and threonine), where their contents were lower. The experimental diets were obtained with the addition of synthetic amino acids (L-Lysine HCL, DL-Methionine, and L-Threonine) to the basal mixture to achieve the values of current recommendations [8] in MMM diet (8.10, 5.78, and 6.90 g/kg DM for lysine, methionine, and threonine, respectively) or the dietary levels that reduced the PUN in a previous trial [9] in MHL diet (8.10, 6.61, and 5.70 g/kg DM for lysine, methionine, and threonine, respectively). A version of the diet, including 5 g/kg DM of alfalfa hay marked with ytterbium, was also manufactured to analyse the true ileal digestibility.

### 2.3. Experimental Procedure

The experimental procedure was conducted over one year (in different batches, 6 in total) under a controlled environment (animals were kept at 15 °C to 22 °C with a photoperiod of 16 h of light and 8 h of darkness). At 28 d of age, 15 weaned rabbits (597 g) were slaughtered with intracardiac puncture with sodium thiopental (75 mg/kg of live weight (LW)) to determine the empty body characteristics at 28 d of age. The rest of the animals (n = 150) were housed in individual cages and assigned to one of the two experimental diets that were received ad libitum until 63 d of age. Mortality and morbidity (presence of diarrhoea) were controlled daily and feed intake and LW were registered weekly. Animals presenting any digestive anomaly, weight loss, or low ingestion were automatically removed from the trial.

At 42 d of age, 28 randomly selected animals were housed in individual metabolic cages of 52 × 44 × 32 cm and, after a week of acclimatisation, a fecal digestibility trial was conducted. From 49 to 53 d of age, feed consumption was controlled, and feces produced were collected. Feces were stored in identified plastic bags and frozen at −20 °C until analysis. Apparent fecal digestibility coefficients for dry matter (DM), crude protein (CP), and gross energy (GE) were determined for each animal. From 53 d of age, 30 randomly selected animals received the same feed marked with ytterbium until their slaughter at 63 d of age. At this age, 55 randomly selected animals (including the previous 30) were weighed and slaughtered with intracardiac injection of sodium thiopental (75 mg kg^−1^LW) between 19:00 to 23:00 h to minimise the influence of caecotrophy on the composition of digestive contents [18]. Samples of ileal content were obtained from the distal part of the small intestine (around 20–40 cm before the ileo-caeco-colic valve) for each animal receiving the market feeds, frozen at −20 °C, freeze-dried, and ground. The whole digestive tract was emptied and reintroduced into the body of the dead animal. Empty bodies, obtained at 28 and 63 d of age (15 and 55 animals, respectively), were weighed and placed in plastic bags, identified, and frozen at −40 °C. Frozen empty bodies were crushed and homogenised in a cutting machine (Tecator, Abusson, France), and one sample per animal was freeze-dried and stored at −40 °C until analysis. True ileal digestibility of CP and amino acids was calculated through estimation of the ileal flow of endogenous nitrogen (based on the previous DM intake) and its amino acid profile, both obtained in growing rabbits [19].

### 2.4. Chemical Analysis

Diets were analysed for DM, ash, CP, neutral detergent fibre (aNDFom), acid detergent fibre (ADFom), lignin (sa), GE, and amino acid content. Feces were analysed for DM, CP, and GE while ileal samples were examined for DM, CP, and amino acid content. Finally, empty body samples were analysed for DM, CP, GE, and amino acid content. Samples were analysed according to the methods of AOAC (2000) [20]: 934.01 for DM, 942.05 for ash, and 976.06 for CP. The aNDFom (assayed with a thermo-stable amylase and expressed exclusive of residual ash), ADFom (expressed exclusive of residual ash), and lignin (determined by solubilisation of cellulose with sulphuric acid, SA) were analysed sequentially as indicated by Van Soest et al. (1991) [21]. The GE determination was done with adiabatic bomb calorimetry (Gallenkamp Autobomb, Loughborough, UK).

The amino acid content was determined after acid hydrolysis with HCL 6N at 110 °C for 23 h as previously described by Bosch et al. (1995) using a Waters (Milford, MA, USA) HPLC system consisting of two pumps (Mod. 515, Waters), an autosampler (Mod. 717, Waters), a fluorescence detector (Mod. 474, Waters), and a temperature control module [22]. Aminobutyric acid was added as internal standard after hydrolysation. The amino acids were derivatised with AQC (6-aminoquinolyl-N-hydroxysuccinimidyl carbamate) and separated with a C-18 reverse-phase column Waters AccQ Tag (150 mm × 3.9 mm). Methionine and cysteine were determined separately as methionine sulphone and cysteic acid, respectively, after performic acid oxidation followed by acid hydrolysis.

### 2.5. Statistical Analysis

From the information obtained from those weaned rabbits slaughtered at 28 d of age (empty body weight and CP, GE, and amino acids contents in the empty body), regression equations were fitted, including LW at 28 d of age as dependent variable for each of them. Values for all these variables at 28 d of age for the animals slaughtered at the end of the retention trial (63 d of age) were estimated for each animal with the cited equations to properly determine nutrient retention during the growing period.

For performance traits (growth, intake, and FCR), digestibility (apparent fecal and true ileal level), and data from each trait was studied using the following model:

$$y_{ijklm}=D_{i}\left|B_{j}\right|W_{k}+p_{l}+e_{ijklm}$$

where *y_ijkl_* represented one record of a given trait; *D_i_* was the effect of diet (two levels: MMM and MHL); *B_j_* was the effect of the Batch (with six levels); *W_k_* was the effect of the Week (with 5 levels); and *p_l_* and *e_ijkl_* were the permanent effect and the random residuals of the records, respectively, considering the lack of homoscedasticity using MIXED model of SAS (Statistical Analyse System) [23].

Average performance traits, digestibility (apparent fecal and true level), and data were studied using the following model:

$$y_{ijkl}=D_{i}|B_{j}+p_{k}+e_{ijkl}$$

where *y_ij_* represented one record of a given trait; *D_i_* was the effect of diet (two levels: MMM and MHL); *B_j_* was the effect of the Batch (with six levels); and *p_l_* and *e_ijkl_* were the permanent effect and the random residuals of the records, respectively, considering the lack of homoscedasticity using GLM model of SAS (Statistical Analyse System) [23]. Some comparisons between slopes were also made using a SAS GLM procedure.

## 3. Results

### 3.1. Digestibility

The effect of the experimental diet on the main coefficients of apparent fecal and ileal digestibility are presented in Table 1. In general, no significant differences in fecal and ileal digestibility between diets were observed except for the coefficient of the ileal apparent digestibility of threonine, which was higher for Diet MMM compared with MHL (+0.14; *p* = 0.0418).

### 3.2. Performance

**Figure 1:**
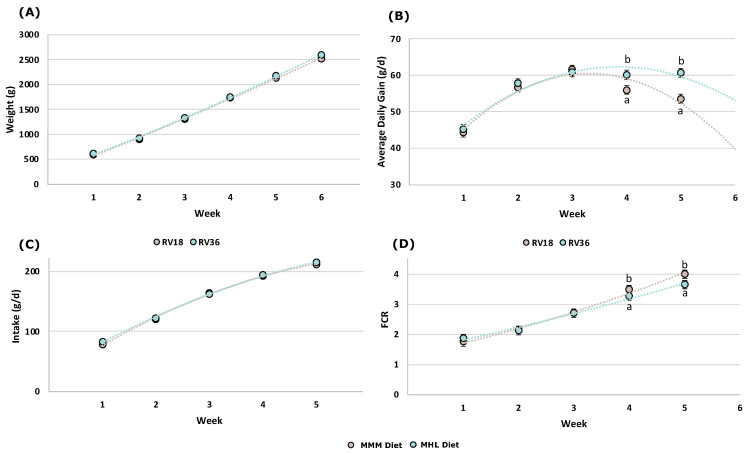
Performance (Weight, Average Daily Gain, Intake and FCR, for Figure 1(**A**–**D**), respectively) of growing rabbits in function of the experimental diets (Least square mean ± standard error). Diet MMM (n = 49), Diet MHL (n = 47). FCR: Feed Conversion Ratio. Week 1 (28–35 d); week 2 (35–42 d); week 3 (42–49 d); week 4 (49–56 d); week 5 (56–63 d). ^a,b^ Means within a row with different letter were significantly different at *p* < 0.05.

Regarding the health status of the rabbits during the fattening period, there were no significant differences in either mortality or morbidity of the rabbits between the dietary treatments, which was, on average, 24.3 and 11.1%, respectively. Figure 1 shows the data on the main performance traits measured in this work. There were no significant differences in daily feed intake (Figure 1C) and LW (Figure 1A) between diets. Nevertheless, animals fed with Diet MHL showed improved global ADG (+2.3 g/d; *p* = 0.0482) and global FCR (−0.10; *p* = 0.0229) than those fed Diet MMM (Figure 1B,D, respectively). These global differences are mainly due to the higher ADG and lower FCR observed in the animals from 49 to 63 d of life (on av. +5.6 g/d; *p* = 0.0482 and −0.29; *p* = 0.0229 respectively), with no significant differences being observed previously.

### 3.3. Retention

Daily energy, protein, and amino acids retained in the empty body of animals with each experimental diet throughout the fattening period are presented in Table 2. There were no significant differences in the retained nutrients between diets.

**Figure 2:**
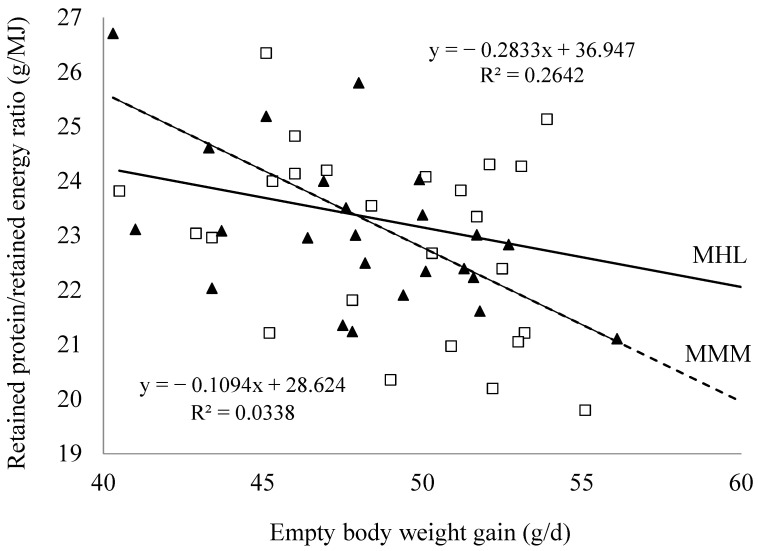
Relationship between daily empty body weight gain with the retained protein to retained energy ratio in the empty body weight gain according to the experimental diet: ▲ MMM and □ MHL.

Finally, Figure 2 shows the relationship between the daily EBWG and the retained protein/energy (g/MJ) ratio obtained for each dietary treatment. In the case of animals fed with Diet MMM, an increase in EBWG decreased the protein/energy ratio retained in the empty body (−0.283 units per unit of EBWG; *p* = 0.0086). However, no significant effect of EBWG on this ratio was observed (*p* = 0.2692) when animals were fed with Diet MHL.

## 4. Discussion

Our initial hypothesis was that the combination of amino acids (Diet MHL) that minimised PUN levels in growing rabbits in a previous study [9] would also be the one that would optimise the protein utilisation of the feed and improve the growth performance traits with respect to the current recommendations (Diet MMM). This improvement in the performance could be due to a hypothetical improvement in the digestive efficiency due to a better amino acid balance. However, no significant differences were found in the apparent fecal digestibility of DM, CP, and GE, nor in the true ileal digestibility of CP and amino acids.

The observed values of true ileal digestibility greater than 100% (on av. 102%) of isoleucine, glutamic acid, and serine in Diet MMM may be due to the fact that the amino acid profile of the endogenous nitrogen used in this work (using values reported in Marín-García et al. (2022) [19]) was richer for these amino acids than reported by Villamide et al. (2013) in adult rabbits (+30%, +28%, and +39%, respectively) [24]. Regarding the amino acids studied in the present work, the true ileal digestibility values were on average 0.79, 0.82, and 0.89 for lysine, sulphur amino acids, and threonine, respectively, slightly lower than those obtained using the endogenous nitrogen profile reported by Villamide et al. (2013) (0.81, 0.88, and 0.92, respectively) [24].

On average, performance traits were like those obtained for this line in previous works [25,26,27]. As was expected, growing rabbits from this paternal line presented high ADG (above 55 g/d) and low FCR (about 2.7), achieving 2.55 kg of LW at 63 d of age. Thus, this population of animals can allow us to correctly test our initial hypothesis; could a diet formulated to minimise animals’ PUN level improve the growth traits of new growing rabbits characterised by a high growth rate? The answer was yes. Growing rabbits fed with Diet MHL showed higher ADG and FCR during the last two weeks of the fattening period than those fed with a diet formulated with the current recommendations (MMM). A reduction in the PUN level could be an indicator of a better fitting of the animal requirements and, consequently, of a better optimisation of the dietary protein, increasing both protein and energy available for growth [27]. The reason why the differences in growth traits were mainly observed at the end of the fattening period may be due to different causes. It may be because the choice of diet that minimised the PUN level was made at 48 d of age [28], this feed being more representative of the amino acid requirements at that age. However, it could also be due to a greater impact of an adequate dietary amino acid formulation on growth at that age. Lean content and lean-to-bone ratio of rabbit carcass increases almost steadily from weeks 1 to 15 d of life.

An increase in ADG is usually associated with an increase in the total amount of protein retained in the empty body [29,30]. However, in the current work, no significant differences between diets were found in the protein retention 9.94 ± 0.28 g/day vs. 9.86 ± 0.28 for Diets MMM and MHL, respectively, nor in the efficiency of dietary protein retention 0.452 ± 0.012 vs. 0.441 ± 0.012 for Diets MMM and MHL, respectively. All of this, despite the higher ADG observed with Diet MHL and the similar composition of EBWG with both diets. These results are mainly due to the following: (i) differences in the ADG of the animals used to measure protein retention were less relevant and non-significant (56.9 ± 1.0 g/day vs. 58.6 ± 0.1.0 g/day for diets MMM and MHL, respectively, *p* = 0.252) than those observed with the total number of the animals, and (ii) particularly, the effect on ADG (and FCR) clearly originated in the last two weeks of the growth period while the protein retention corresponds to the entire growth period, logically diluting a probable effect of the diet on protein retention during the last two weeks of the growth period. Nevertheless, animals fed with Diet MMM reduced the protein-to-energy ratio in the EBWG as the growth rate increased, as previously observed by Marín-García et al. (2020a) [5] with a similar diet, while this ratio did not depend on growth rate in animals fed with Diet MHL, which indicates a better utilisation of its protein in animals with a higher growth potential and that its lysine, sulphur amino acids, and threonine levels are more suitable for them. These levels would be particularly recommended in the last two weeks of the growing period, although more studies would be needed in this regard.

In view of these results, it could be interesting to assess and compare the current lysine, sulphur amino acids, and threonine recommendations [8] with their respective levels in Diet MHL at total, as well as the apparent and true ileal contents, to provide more accurate information to formulate diets for growing rabbits (Table 3). In the case of lysine, both diets had the same levels (8.1 g/kg DM). This value is quite like those recommended by other authors [31,32], although higher than the 6.7 g/kg DM proposed [33]. The improved growth performance traits obtained in this work with higher dietary sulphur amino acid levels (6.6 g/kg DM) compared with current recommendations (5.7 g/kg DM) have already been observed by other authors [32,33,34,35], who obtained greater performance with sulphur amino acid levels from 6.7 to 8.0 g/kg DM. All these data are closer to MHL values than MMM ones, which could be indicating a possible underestimation of the current sulphur amino acids recommendations for growing rabbits.

Finally, in the case of dietary threonine, studies about its requirements are variable. The values of the current recommendations for MMM (6.9 g/kg DM) are closer to those proposed by NRC (1977) [33] and de Blas et al. (1998) (6.7 g/kg DM) [36]. Nevertheless, Adamson and Fisher (1973) [32] proposed lower values for the threonine (5.6 g/kg DM) quite like the current updated proposal (5.7 g/kg DM). A possible explanation for the lack of consensus in threonine recommendations may be both in the contribution of caecotrophy or to possible interactions with other amino acids in the diet, such as glycine. It must be considered that the contribution of soft feces to the total threonine intake is the highest of all amino acids [37], making it possible to lessen their level of inclusion in the diet.

## 5. Conclusions

In view of the results, it can be concluded that, compared to the current recommendations, the lysine, sulphur amino acids, and threonine levels in Diet MHL allow an improvement of ADG and FCR in growing rabbits of a paternal line, showing better dietary protein utilisation in animals with higher growth potential. Consequently, new recommended amino acid levels are proposed: 8.1, 6.6, and 5.7 g/kg DM of total lysine, sulphur amino acids, and threonine, respectively; 5.2, 4.2, and 2.9 g/kg DM of apparent ileal digestible lysine, sulphur amino acids, and threonine, respectively; or 6.4, 5.4, and 5.0 g/kg DM of true ileal digestible lysine, sulphur amino acids, and threonine, respectively.

## Figures and Tables

**Table 1:** Coefficients of apparent fecal (n = 28) and ileal (n = 30) digestibility for some nutrients in function of the experimental diet during the fattening period (Least square mean ± standard error).

	MMM		MHL		*p*-Value
Fecal coefficients: (49 to 53 d old)					
Dry matter	0.547	±0.006	0.533	±0.005	0.0770
Crude protein	0.733	±0.007	0.731	±0.008	0.1122
Gross energy	0.606	±0.005	0.599	±0.005	0.2806
*Apparent Ileal coefficients*: (63 d old)					
Dry matter	0.395	±0.025	0.439	±0.028	0.2577
Crude protein	0.576	±0.036	0.556	±0.037	0.6957
Alanine	0.591	±0.046	0.552	±0.052	0.5612
Arginine	0.805	±0.027	0.753	±0.031	0.2373
Aspartic acid	0.618	±0.049	0.561	±0.057	0.4548
Cysteine	0.416	±0.052	0.552	±0.061	0.1064
Glutamic acid	0.781	±0.033	0.731	±0.038	0.3151
Glycine	0.344	±0.052	0.339	±0.064	0.9575
Histidine	0.753	±0.033	0.684	±0.038	0.1761
Isoleucine	0.658	±0.049	0.596	±0.056	0.4048
Leucine	0.664	±0.049	0.606	±0.055	0.4234
Lysine	0.708	±0.050	0.600	±0.058	0.1541
Methionine	0.741	±0.027	0.740	±0.034	0.9795
Phenylalanine	0.691	±0.044	0.635	±0.051	0.4014
Proline	0.678	±0.029	0.690	±0.033	0.7828
Serine	0.521	±0.041	0.461	±0.060	0.4440
Threonine	0.645	±0.042	0.507	±0.048	0.0408
Tyrosine	0.549	±0.050	0.505	±0.057	0.5667
Valine	0.622	±0.045	0.578	±0.050	0.5106
*True Ileal coefficients*: (63 d old)					
Crude protein	0.807	±0.036	0.786	±0.040	0.7059
Alanine	0.860	±0.046	0.792	±0.053	0.3376
Arginine	0.849	±0.033	0.763	±0.038	0.0988
Aspartic acid	0.874	±0.049	0.783	±0.057	0.2372
Cysteine	0.790	±0.052	0.806	±0.061	0.8411
Glutamic acid	1.016	±0.033	0.952	±0.038	0.2180
Glycine	0.669	±0.055	0.620	±0.064	0.5616
Histidine	0.870	±0.033	0.792	±0.038	0.1271
Isoleucine	1.033	±0.050	0.938	±0.058	0.2234
Leucine	0.866	±0.049	0.773	±0.057	0.2256
Lysine	0.854	±0.050	0.721	±0.058	0.0967
Methionine	0.851	±0.029	0.812	±0.034	0.4494
Phenylalanine	0.844	±0.044	0.757	±0.051	0.2156
Proline	0.938	±0.029	0.904	±0.033	0.4551
Serine	1.025	±0.041	0.936	±0.060	0.2765
Threonine	0.839	±0.049	0.931	±0.043	0.1689
Tyrosine	0.711	±0.050	0.646	±0.057	0.4064
Valine	0.922	±0.045	0.848	±0.052	0.2919

**Table 2:** Retained energy (kJ), protein (g), and amino acids (g) per g of empty body weight gain (EBWG) during the growing period for each experimental diet (n = 55) (Least square mean ± standard error).

	MMM		MHL		*p*-Value
Crude protein intake (g/day)	22.6	±0.5	23.1	±0.5	0.4484
EBWG (g/day)	47.2	±0.9	48.6	±0.9	0.2735
Energy	8.999	±0.239	8.679	±0.239	0.3493
Protein	0.2104	±0.0043	0.2024	±0.0043	0.1898
Alanine	0.0099	±0.0003	0.0096	±0.0003	0.3712
Arginine	0.0147	±0.0005	0.0150	±0.0005	0.7518
Aspartic acid	0.0150	±0.0005	0.0148	±0.0005	0.8158
Cysteine	0.0036	±0.0002	0.0035	±0.0002	0.6560
Glutamic acid	0.0241	±0.0008	0.0247	±0.0008	0.6029
Glycine	0.0170	±0.0004	0.0161	±0.0004	0.1516
Histidine	0.0040	±0.0002	0.0040	±0.0003	0.9822
Isoleucine	0.0069	±0.0001	0.0069	±0.0001	0.9041
Leucine	0.0138	±0.0003	0.0138	±0.0003	0.8959
Lysine	0.0121	±0.0004	0.0120	±0.0004	0.9501
Methionine	0.0040	±0.0001	0.0038	±0.0001	0.1526
Phenylalanine	0.0069	±0.0002	0.0069	±0.0002	0.9542
Proline	0.0097	±0.0003	0.0096	±0.0003	0.7890
Serine	0.0086	±0.0003	0.0089	±0.0003	0.5548
Threonine	0.0074	±0.0003	0.0077	±0.0002	0.4507
Tyrosine	0.0055	±0.0003	0.0057	±0.0003	0.5733
Valine	0.0093	±0.0002	0.0094	±0.0002	0.8986

**Table 3:** Current and new recommendations (g/kg dry matter) for the first three limiting amino acids in growing rabbits.

	Current (MMM)			New (MHL)		
	Lysine	Sulphur Amino Acids	Threonine	Lysine	Sulphur Amino Acids	Threonine
Total	8.1 ^1^	5.8 ^1^	6.9 ^1^	8.1 ^3^	6.6 ^3^	5.7 ^3^
Apparent fecal digestible	6.3 ^1^	4.4 ^1^	4.8 ^1^			
Apparent ileal digestible	5.2 ^2^	3.6 ^2^	4.5 ^2^	5.2 ^2^	4.2 ^2^	2.9 ^2^
True ileal digestible	6.4 ^2^	4.7 ^2^	6.1 ^2^	6.4 ^2^	5.4 ^2^	5.0 ^2^

^1^ Current recommendations [8]. ^2^ Data obtained in the present work. ^3^ Data proposed by Marín-García et al. (2020c) to minimize the plasma urea nitrogen level [9]. All data have been analyzed in the present work.

## Data Availability

The datasets of the current study are available from the corresponding author upon reasonable request.

## Appendix A

**Table A1:** Ingredients (g/kg) and chemical composition (g/kg dry matter) of the basal mixture and the experimental diets.

	Basal Mixture	MMM	MHL
Ingredients			
Wheat bran	300		
Straw	175		
Sunflower meal	167		
Alfalfa hay	148		
Beet pulp	120		
Barley	42		
Beet molasses	29		
Palm oil	4.5		
Sodium chloride	4.8		
Calcium carbonate	2.6		
L-Lysine HCL	2.4		
Monocalcium phosphate	0.2		
Vitamin-trace element mixture ^1^	5.2		
*Chemical composition*			
Dry matter (DM; g/kg)	896	929	921
Ashes	73.2	70.7	71.2
Crude protein	157	155	157
Gross Energy (MJ/kg DM)	-	18.01	18.36
Ether extract	26.7	25.8	26.0
Neutral detergent fiber	473	456	460
Acid detergent fiber	275	263	267
Acid detergent lignin	58.1	56.1	56.4
Digestible Energy ^2^ (MJ/kg DM)	-	10.85	10.98
Digestible Protein ^2^	-	113	115
Digestible Protein/Digestible Energy	-	10.41	10.47
Amino acids: Alanine	5.81	5.61	5.62
Arginine	8.25	7.96	8.03
Aspartic Acid	12.7	12.2	12.4
Cysteine (Cys)	2.36	2.28	2.30
Glutamic acid	26.7	24.8	26.3
Glycine	7.43	7.27	7.23
Histidine	3.02	2.91	2.94
Isoleucine	5.42	5.32	5.27
Leucine	9.59	9.25	9.33
Lysine	7.29	8.10 ^3^	8.10 ^3^
Methionine (Met)	2.64	3.50 ^4^	4.31 ^4^
Phenylalanine	6.07	5.85	5.91
Proline	8.44	8.14	8.21
Serine	6.63	6.25	6.47
Sulphur (Met + Cys)	4.97	5.78	6.61
Threonine	5.04	6.90 ^5^	5.70 ^5^
Tyrosine	2.99	2.88	2.99
Valine	7.42	7.16	7.23

^1^ Contains per kg of feed: vitamin A: 8375 IU; vitamin D3: 750 IU; vitamin E: 20 mg; Vitamin K3: 1 mg; vitamin B1: 1 mg; vitamin B2: 2 mg; vitamin B6: 1 mg; nicotinic acid: 20 mg; choline chloride: 250 mg; magnesium: 290 mg; manganese: 20 mg; zinc: 60 mg; iodine: 1.25 mg; iron: 26 mg; copper: 10 mg; cobalt: 0.7 mg; butyl hydroxylanysole and ethoxiquin mixture: 4 mg. ^2^ Experimentally determined according to Perez et al. (1995) [38]. Using 14 helthy growing rabbits of 49 d of live per diet weaned at 28 d. Feces were collected during 4 d after a period of 7 d of adaptation to diets. ^3,4,5^ Values were obtained by adding L-Lysine HCL, DL-Methionine and L-Threonine, respectively.
